# Interactive effect of acute and chronic glycemic indexes for severity in acute ischemic stroke patients

**DOI:** 10.1186/s12883-018-1109-1

**Published:** 2018-08-03

**Authors:** Keon-Joo Lee, Ji Sung Lee, Keun-Hwa Jung

**Affiliations:** 1 Department of Neurology, Seoul National University Bundang Hospital, Seoul, South Korea; 2 Clinical Research Center, Asan Medical Center, Seoul, South Korea; 3 Department of Neurology, Seoul National University Hospital, 101, Daehangno, Jongno-gu, Seoul, 03080 South Korea

**Keywords:** Glucose, Ischemic stroke, Etiology, Hemoglobin A1c, Hyperglycemia

## Abstract

**Background:**

Diabetes mellitus is a well-established risk factor for ischemic stroke and is known to increase stroke risk by 2–6 fold. Numerous studies have reported the relationship between parameters for glycemic status and stroke-related outcomes; however, studies focusing on the interaction between acute and chronic glycemic status indexes with stroke phenotype are lacking.

**Methods:**

Acute ischemic stroke patients who were admitted to a tertiary hospital stroke center from 2002 to 2015 were consecutively enrolled in this study. Fasting blood sugar (FBS) and serum glycated hemoglobin (HbA1c) levels were recorded as acute and chronic glycemic indexes, respectively. The associations between initial stroke severity and both glycemic indexes were evaluated with consideration of the interaction between the glycemic indexes. Moreover, the distinct effects of stroke subtypes were evaluated.

**Results:**

A total of 2595 patients were included in the final analysis. After adjustment for covariates, FBS was associated with initial stroke severity (*P* < 0.001), while HbA1c was not (*P* = 0.16). However, an interaction between FBS and HbA1c in association with initial stroke severity was observed (*P* < 0.001). The association between FBS and initial stroke severity was stronger, with a relatively normal HbA1c level. Among stroke subtypes, the interactions were significant for the large artery disease and cardioembolism subtypes (all, *P* < 0.001), but for the small vessel occlusion subtype (*P* = 0.63).

**Conclusions:**

This study shows that HbA1c is an effect modifier for the association between FBS and initial stroke severity, and the interactive effect differs among stroke subtypes.

**Electronic supplementary material:**

The online version of this article (10.1186/s12883-018-1109-1) contains supplementary material, which is available to authorized users.

## Background

Diabetes mellitus is an established modifiable risk factor for ischemic stroke, which accounts for approximately 3–20% of stroke risk [1, 2]. The risk of stroke is 2–6 times higher in diabetes patients than in non-diabetic individuals [3]. In the acute stroke stage, glycemic parameters, such as fasting blood sugar (FBS) or serum glycated hemoglobin (HbA1c), are known to be related to post-stroke outcomes, and the current guideline recommends strict glycemic control (normoglycemia) for the management of acute ischemic stroke [4].

However, results that focus on the simultaneous or interactive effects for the two glycemic indexes, representing immediate changes in the glycemic status at the acute stage of ischemic stroke and previously cumulative changes in the glycemic status (HbA1c), are limited. The impact of glucose level in the acute stage of ischemic stroke might vary between different HbA1c statuses. Moreover, as ischemic stroke is a heterogenous disease entity, the effect of glycemic status might differ among stroke subtypes with distinct pathophysiological mechanisms [3, 5]. Estimating the effect of acute glycemic status based on different underlying conditions would be useful for optimizing care in acute stroke patients.

This study investigated the interaction between the indexes for acute and chronic glycemic status and examined whether the effects differed among stroke subtypes.

## Methods

### Study subjects

Acute ischemic stroke patients who were admitted to a tertiary hospital between January 2002 and May 2015 were included according to the following eligibility criteria (Additional file 1: Figure S1): 1) age older than 18 years, 2) relevant ischemic lesion confirmed with brain imaging (computed tomography or magnetic resonance imaging), 3) admission within 7 days of symptom onset, and 4) clearly determined stroke subtype of large artery disease (LAD), small vessel occlusion (SVO), or cardioembolism (CE). Stroke subtypes were classified according to the TOAST classification [5] and were determined at the time of the patient’s discharge via consensus between at least two trained neurologists. Medical history and results of work-ups during hospitalization (e.g., cerebral angiography, transcranial Doppler sonography, transthoracic and transesophageal echocardiography, electrocardiogram, and 24-h Holter monitoring) were reviewed for subtype determination. The patients with 1) missing glycemic status indicators, 2) an unclear previous diagnosis of diabetes mellitus, and 3) missing outcome data were excluded.

### Data collection

Demographic data and clinical parameters, namely age, sex, body mass index, time from symptom onset to hospital arrival, hyperacute reperfusion therapy administration, antithrombotic use at the acute stage, risk factor profiles including previous history, initial blood pressure, and lipid panel results at admission, were collected.

Glycemic indexes, which use the FBS level as an indicator for the acute glycemic status and glycated hemoglobin index (HbA1c) as an indicator for the chronic status, were measured in each subject after at least 8 h of fasting on the first or second day of admission according to an institutional protocol of blood sampling [6]. FBS was selected instead of the initial glucose level to minimize the effect of meals [7].

The outcome parameters included the National Institute of Health Stroke scale (NIHSS) score measured at hospital arrival, which represented initial stroke severity, and the modified Rankin scale (mRS) scores, which represented functional status at discharge [8]. These stroke scales were measured and recorded by the attending neurologist at both the times of hospital arrival and discharge.

The study design and subject data collection were approved by the Institutional Review Board.

### Statistical analysis

The characteristics of study subjects are described as numbers and percentages for categorical variables and as mean ± standard deviation for interval variables. Stroke scale scores and interval between stroke onset and hospital arrival are presented as medians and interquartile ranges.

A bivariate correlation analysis between the two glycemic indexes (FBS and HbA1c) was performed using Spearman’s rank correlation coefficient. FBS and HbA1c were centered for the arithmetic mean of each parameter, and the interaction terms of the centered FBS and HbA1c (FBS*HbA1c) were introduced into a multivariable linear regression model along with predetermined covariates (age, sex, interval between stroke onset and hospital arrival, body-mass index, hypertension, diabetes, hyperlipidemia, heart disease, previous stroke history, smoking, stroke subtype, systolic and diastolic blood pressure, LDL cholesterol, HDL cholesterol, and triglyceride level) and the centered glycemic indexes to examine their associations with the admission NIHSS scores.

To evaluate the association between the discharge mRS score and the glycemic indexes, a shift analysis using a multivariable ordinal regression model and an analysis for mRS scores dichotomized into good (mRS scores: 0–1) and poor (mRS scores: 2–6) using a multivariable binary logistic regression model were performed. In these analyses, the NIHSS score at admission along with additional covariates, including the variables indicating acute treatment status (acute antithrombotics administration and hyperacute reperfusion therapy), were included in the model in order to evaluate whether the glycemic indexes had a secondary effect on patient outcome independently of their effect on initial stroke severity (Additional file 2: Table S1).

**Fig. 1:**
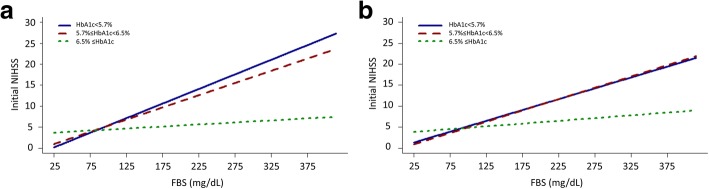
Plots for interaction between fasting blood sugar, HbA1c, and initial stroke severity. **a** unadjusted and (**b**) adjusted plots for interactions. FBS, fasting blood sugar; NIHSS, National Institute of Health Stroke Scale score

**Fig. 2:**
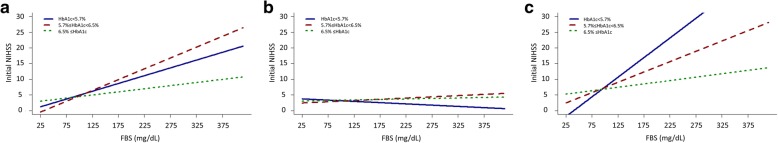
Adjusted plots for interaction among stroke subtypes. **a** large artery disease, **b** small vessel occlusion, and (**c**) cardioembolism. FBS, fasting blood sugar; NIHSS, National Institute of Health Stroke Scale score

**Fig. 3:**
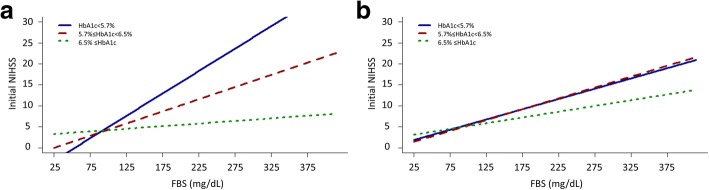
Adjusted plots for interaction by history of diabetes. **a** patients with previous history of diabetes, and (**b**) no history of diabetes. FBS, fasting blood sugar; NIHSS, National Institute of Health Stroke Scale score

Linear fit line from scatter plots showing the estimated correlation between FBS and outcome measurements according to HbA1c levels in the normal (HbA1c < 5.7%), pre-diabetes (5.8% ≤ HbA1c < 6.5%), and diabetes (6.5% ≤ HbA1c) range was presented in figures (Figs. 1, 2 and 3) [6].

In order to evaluate the interaction between glycemic indexes and stroke subtypes, the interactions between the original variables and every combination of terms for each variable (FBS*HbA1c, FBS*stroke subtypes, HbA1c*stroke subtypes, and FBS*HbA1c*stroke subtypes) were introduced into the model (Additional file 2: Table S2).

As an interaction between glycemic indexes and stroke subtypes was demonstrated to exist, a subgroup analysis among the three subtypes was performed and an additional subgroup analysis of the relationship between patients who had a previous history of diabetes and those who had not was conducted at the post-hoc level.

Two-tailed *P* values < 0.05 were considered statistically significant. All statistical analyses were performed with the use of R software, version 3.4 (R Foundation for Statistical Computing, Vienna, Austria).

## Results

A total of 2595 patients were included in the final analysis (Additional file 1: Figure S1). The basic characteristics of the study subjects are presented in Table 1. There were 1047 (40.3%) patients classified as LAD, 832 (32.1%) as SVO, and the remaining 716 (27.6%) as CE. The mean age of the subjects was 66.9 ± 11.9 years, and most patients (61.4%) were male. Thirty-five percent of the patients were previously diagnosed with diabetes. The median time to hospital arrival was approximately 17 h. The median NIHSS score at arrival was 3, and 7.3% of patients received hyperacute reperfusion therapy.

**Table 1:** Baseline characteristics of all study subjects (*N* = 2595)

	Values
Age	66.9 ± 11.9
Male	1593 (61.4)
Time to hospital arrival (hours)	17 (4–49)
Body-mass index (kg/m^2^)	23.86 ± 3.30
Hypertension	1821 (70.2)
Diabetes	918 (35.4)
Hyperlipidemia	688 (26.5)
Smoking	881 (33.9)
Heart disease	632 (24.4)
Previous stroke	550 (21.2)
Reperfusion therapy	189 (7.3)
Acute medication	
Antiplatelet	2081 (80.2)
Anticoagulation	511 (19.7)
Stroke subtype	
Large artery disease	1047 (40.3)
Small vessel occlusion	832 (32.1)
Cardioembolism	716 (27.6)
Initial NIHSS	3 (1–6)
Discharge mRS	2 (2–4)
SBP (mmHg)	153.3 ± 27.3
DBP (mmHg)	85.6 ± 15.6
Total cholesterol level (mg/dL)	177.3 ± 40.0
LDL cholesterol level (mg/dL)	107.6 ± 35.2
HDL cholesterol level (mg/dL)	45.1 ± 12.8
Triglyceride (mg/dL)	123.8 ± 72.3
Fasting blood sugar (mg/dL)	112.8 ± 40.5
HbA1c (%)	6.4 ± 1.3

*NIHSS* National Institute of Health Stroke Scale score, *mRS* modified Rankin’s scale, *SBP* systolic blood pressure, *DBP* diastolic blood pressure, *LDL* low-density lipoprotein, *HDL* high density-lipoprotein

FBS and HbA1c were moderately positively correlated (ρ = 0.52, *P* < 0.001; Additional file 3: Figure S2). Thus, it was necessary to introduce an interaction term in further analysis models. When FB and HbA1c and their interaction term (FBS*HbA1c) were inputted to a multivariable linear regression model, FBS (*P* < 0.001), but not HbA1c (*P* = 0.16), was shown to have an association with the initial NIHSS score (Table 2). Moreover, these two glycemic indexes had an interaction (*P* < 0.001) regarding their effect on initial stroke severity. The interaction plot displayed in Fig. 1 shows the estimated correlation between FBS and initial NIHSS scores in different HbA1c ranges. The correlations appeared stronger when HbA1c was within a relatively normal range than when it was higher for both unadjusted (A) and adjusted (B) models.

**Table 2:** Association between glycemic parameters and initial stroke severity, considering interactions

Variables	Coefficient (B)	Standard error (ε)	*t*-value	*P*-value
FBS (mg/dL)	0.03	0.004	9.81	< 0.001
HbA1c (%)	−0.18	0.12	− 1.42	0.16
FBS * HbA1c	−0.007	0.001	−4.81	< 0.001
Age (y)	0.040	0.009	4.28	< 0.001
Sex	0.33	0.24	1.41	0.16
Stroke subtype				
LAD (reference)	–	–	–	–
SVO	−1.64	0.24	−6.93	< 0.001
CE	1.90	0.34	5.60	< 0.001
Time to hospital arrival (hour)	−0.01	0.003	−5.38	< 0.001
Body-mass index	−0.16	0.032	−5.12	< 0.001
Hypertension	−0.30	0.23	−1.28	0.20
Diabetes	−0.78	0.30	−2.60	0.009
Hyperlipidemia	−0.21	0.24	−0.90	0.37
Heart disease	0.24	0.33	0.73	0.47
Previous stroke history	0.74	0.25	3.01	0.003
Smoking	−0.03	0.24	−0.11	0.92
SBP (mmHg)	−0.007	0.005	−1.42	0.16
DBP (mmHg)	0.02	0.009	2.51	0.01
LDL cholesterol (mg/dL)	0.007	0.0	2.27	0.02
HDL cholesterol (mg/dL)	−0.03	0.008	−3.78	< 0.001
Triglyceride (mg/dL)	−0.002	0.002	−1.26	0.21

*FBS* fasting blood sugar, *LAD* large artery disease, *SVO* small vessel occlusion, *CE* cardioembolism, *SBP* systolic blood pressure, *DBP*diastolic blood pressure, *LDL* low-density lipoprotein, *HDL* high-density lipoprotein

There was an interaction between the glycemic indexes for the mRS score at discharge in the shift analysis (*P* = 0.04). However, neither an association nor an interaction between the glycemic indexes and the functional outcome at discharge was shown when the initial NIHSS scores were included in the models (Additional file 2: Table S1).

There was an interaction between the stroke subtypes and the glycemic indexes and their interaction term (Additional file 2: Table S2); thus, a subgroup analysis for stroke subtypes was performed (Table 3 and Fig. 2). FBS and the interaction term, FBS*HbA1c, were shown to be associated with the initial NIHSS score in the LAD and CE subtypes (all *P* < 0.001), while the association was absent in the SVO subtype (Table 3). The linear fit lines from the scatter plots showed a stronger correlation in patients with an HbA1c range lower than 6.5% in the LAD and CE subtypes (Fig. 2).

**Table 3:** Interaction between glycemic parameters and initial stroke severity among stroke subtypes

	Coefficient (B)	Standard error (ε)	*t*-value	*P*-value
Large artery disease (*N* = 1047)				
FBS (mg/dL)	0.05	0.006	8.47	< 0.001
HbA1c (%)	−0.25	0.18	−1.34	0.18
FBS * HbA1c	−0.01	0.002	−4.89	< 0.001
Small vessel occlusion (*N* = 832)				
FBS (mg/dL)	0.02	0.003	0.65	0.52
HbA1c (%)	0.04	0.12	0.34	0.74
FBS * HbA1c	0.0006	0.001	0.48	0.63
Cardioembolism (*N* = 716)				
FBS (mg/dL)	0.06	0.009	6.91	< 0.001
HbA1c (%)	−0.66	0.33	−1.96	0.05
FBS * HbA1c	−0.02	0.005	−3.36	< 0.001

*FBS* fasting blood sugar

Adjusted for age, sex, time before hospital arrival, body-mass index, hypertension, diabetes, hyperlipidemia, heart disease, previous stroke history, smoking, systolic and diastolic blood pressure, LDL cholesterol, HDL cholesterol and triglyceride level

In the post-hoc analysis, in which the patients were categorized based on the diagnosis of diabetes before admission, the interaction between the glycemic indexes was significant regardless of diabetes history (Table 4 and Fig. 3). However, the HbA1c level was not negatively correlated with initial stroke severity in patients previously diagnosed with diabetes (*B* = − 0.26, *P* = 0.03).

**Table 4:** Interaction between glycemic parameters for initial stroke severity based on history of diabetes

	Coefficient (B)	Standard error (ε)	*t*-value	*P*-value
Diabetes (*N* = 918)				
FBS (mg/dL)	0.02	0.003	6.18	< 0.001
HbA1c (%)	−0.26	0.12	−2.19	0.03
FBS * HbA1c	−0.006	0.002	−3.43	< 0.001
Non-diabetes (*N* = 1677)				
FBS (mg/dL)	0.06	0.007	8.56	< 0.001
HbA1c (%)	−0.53	0.31	−1.71	0.09
FBS * HbA1c	−0.23	0.0009	−2.45	0.02

*FBS* fasting blood sugar

Adjusted for age, sex, time to hospital arrival, body-mass index, stroke subtype, hypertension, hyperlipidemia, heart disease, previous stroke history, smoking, systolic and diastolic blood pressure, LDL cholesterol, HDL cholesterol and triglyceride level

## Discussion

In this study, we demonstrated that FBS and HbA1c interact with initial stroke severity in acute ischemic stroke patients. The chronic glycemic status, represented by the HbA1c level, seemed to modify the effect of acute glycemic status on stroke severity. Moreover, this effect differed among stroke subtypes in that the interaction was significant in patients with the LAD and CE subtypes, while insignificant in those with the SVO subtype.

Numerous prior studies have shown the association between glucose levels in the acute stage and outcomes in ischemic stroke patients. Higher blood glucose level on admission is known to be correlated with stroke progression [9, 10], poor functional outcomes [9, 11], and mortality [12, 13]. In addition, high blood glucose levels have been associated with poor outcomes after reperfusion therapy [14–16] or hemorrhagic transformation following initial ischemic stroke occurrence [17]. Another glycemic index, HbA1c, is known to represent glycemic control status within the past 2–3 months [18], but relatively few studies have addressed the effect of HbA1c on stroke outcomes. For example, one study involving patients from the Fukuoka stroke registry showed that HbA1c predicts neurological deterioration and functional outcome at discharge [19], while another observational study did not find an association between HbA1c and functional outcomes [20].

To date, no study has considered the interactive effect of these two glycemic indexes. A recent study by Chinese investigators showed that newly diagnosed diabetes with isolated elevation of HbA1c that was not accompanied by elevated blood glucose level was not associated with poor outcome after ischemic stroke [21]. This result implies that the glycemic status before a stroke might have a different effect than the acute glycemic status.

Several pathophysiological mechanisms have been proposed regarding how a high glucose level may result in poor outcomes after ischemic stroke. Notably, reperfusion injury is suggested to be a mainstay of the harmful effect in which hyperglycemia would augment oxidative stress [22]. According to this background, the current clinical guideline recommends avoiding hyperglycemia and instead maintaining normoglycemia within the range of 140 to 180 mg/dL during the acute stage of ischemic stroke [4]. In addition, a high glucose level is known to compromise the recruitment of collateral circulation in ischemic stroke animal models or clinical studies [23, 24]. The lack of association in the SVO subtype might be explained by a relatively small infarct size and lesser influence of the collateral circulation due to the different pathophysiology of this subtype [25]. However, considering that the hyperglycemic status supplies sufficient glucose and energy to the brain, the effect of hyperglycemia seems to be complicated in the ischemic brain [22]. Our result, which showed a weaker association of acute hyperglycemia with stroke severity in patients with higher HbA1c levels, may be an implication of this beneficial effect of hyperglycemia.

Because the glycemic indicators in our study were measured after stroke onset, the bi-directional effect of glycemic change and stroke should be considered. The increase in glucose level during the acute stroke period is sometimes noted with the term “stress hyperglycemia,” which results partly from an elevated sympathetic tone [22, 26]. Stress hyperglycemia might be a marker of impaired glucose regulation in patients with insulin resistance and is known to be associated with poor outcome after stroke [26, 27]. The association between FBS level and stroke severity in the patients of our study with a relatively normal HbA1c might indicate the effect of stress hyperglycemia; however, stress hyperglycemia might be understood as a protective response that may help survival [28]. The pathophysiological or clinical implications of stress hyperglycemia should be revealed by future studies.

The association between FBS and stroke severity was weaker when the HbA1c level was higher. The patients with higher HbA1c level had several other cardiovascular risk factors and were using medication including antidiabetic agents, statins, and antithrombotics. Considering that such agents are potentially beneficial for protecting the brain against ischemic insult [29–33], the effect of FBS on stroke severity in those patients might be attenuated. However, when we conducted a post-hoc subgroup analysis comparing the patients who were previously diagnosed with diabetes with those who were not, the noted interaction between the glycemic indexes remained valid in both groups (Table 4 and Fig. 3).

There are several limitations to our study. First, as discussed above, a reversed temporal relationship between the measurements of the glycemic indexes and the stroke severity scale should be considered, and a causal relationship could not be determined from this study. Second, the outcome measurements in our study consisted of stroke scales, which might not directly implicate the pathological status of the study subjects [8]. Parameters derived from brain images, such as infarct volume, might be more useful in this respect. Third, information on medications prior to stroke was not collected, although prior antithrombotics, statins, and some oral hypoglycemic agents may influence the stroke phenotype. Fourth, the period of patient enrollment spanned more than a decade, and changes in clinical practice during this period would be considerable.

## Conclusion

Our study results showed that HbA1c was an effect modifier for the association between FBS and stroke severity and that stroke subtypes affected the intensity of the association. Further studies are warranted for evaluating the pathophysiological aspects of these findings and their implications for acute stroke management.

## Additional files

Additional file 1: **Figure S1.** Eligibility criteria of the study subjects. (TIF 86 kb)

Additional file 2: **Table S1.** Interaction between glycemic parameters for functional outcome (modified Rankin’s scale) at discharge. **Table S2.** Interaction between glycemic parameters and stroke subtypes for initial stroke severity. (DOCX 25 kb)

Additional file 3: **Figure S2.** Correlation plot between HbA1c and fasting blood sugar. (TIF 72 kb)

## Abbreviations

CE: Cardioembolism
FBS: Fasting blood sugar
HbA1c: Serum glycated hemoglobin
HDL: High density lipoprotein
LAD: Large artery disease
LDL: Low density lipoprotein
mRS: Modified Rankin scale
NIHSS: National Institute of Health Stroke scale
SVO: Small vessel occlusion
TOAST: Trial of Org 10,172 in Acute Stroke Treatment

## References

[1] Donnell MJO, Chin SL, Rangarajan S, Xavier D, Liu L, Zhang H (2016). Global and regional eff ects of potentially modifiable risk factors associated with acute stroke in 32 countries ( INTERSTROKE ): a case-control study. Lancet.

[2] Willey JZ, Moon YP, Kahn E, Rodriguez CJ, Rundek T, Cheung K (2014). Population attributable risks of hypertension and diabetes for cardiovascular disease and stroke in the northern Manhattan study. J Am Heart Assoc.

[3] Sander D, Kearney MT (2009). Reducing the risk of stroke in type 2 diabetes: pathophysiological and therapeutic perspectives. J Neurol.

[4] Jauch EC, Saver JL, Adams HP, Bruno A, Connors JJ, Demaerschalk BM (2013). Guidelines for the early management of patients with acute ischemic stroke: a guideline for healthcare professionals from the American Heart Association/American Stroke Association. Stroke.

[5] Adams HP, Bendixen BH, Kappelle LJ, Biller J, Love BB, Gordon DL (1993). Classification of subtype of acute ischemic stroke. Definitions for use in a multicenter clinical trial. TOAST. Trial of org 10172 in acute stroke treatment. Stroke.

[6] American Diabetes Association (2018). 2. Classification and Diagnosis of Diabetes: Standards of Medical Care in Diabetes—2018. Diabetes Care.

[7] Moebus S, Göres L, Lösch C, Jöckel KH (2011). Impact of time since last caloric intake on blood glucose levels. Eur J Epidemiol.

[8] Kasner SE (2006). Clinical interpretation and use of stroke scales. Lancet Neurol.

[9] Baird TA, Parsons MW, Phanh T, Butcher KS, Desmond PM, Tress BM (2003). Persistent poststroke hyperglycemia is independently associated with infarct expansion and worse clinical outcome. Stroke.

[10] Shimoyama T, Kimura K, Uemura J, Saji N, Shibazaki K (2014). Elevated glucose level adversely affects infarct volume growth and neurological deterioration in non-diabetic stroke patients, but not diabetic stroke patients. Eur J Neurol.

[11] Ntaios G, Egli M, Faouzi M, Michel P (2010). J-shaped association between serum glucose and functional outcome in acute ischemic stroke. Stroke.

[12] Hyvärinen M, Qiao Q, Tuomilehto J, Laatikainen T, Heine RJ, Stehouwer CD (2009). Hyperglycemia and stroke mortality: comparison between fasting and 2-h glucose criteria. Diabetes Care.

[13] Williams LS, Rotich J, Qi R, Fineberg N, Espay A, Bruno A (2002). Effects of admission hyperglycemia on mortality and costs in acute ischemic stroke. Neurology.

[14] Alvarez-Sabín J, Molina CA, Montaner J, Arenillas JF, Huertas R, Ribo M (2003). Effects of admission hyperglycemia on stroke outcome in reperfused tissue plasminogen activator-treated patients. Stroke.

[15] Poppe AY, Majumdar SR, Jeerakathil T, Ghali W, Buchan AM, Hill MD (2009). Admission hyperglycemia predicts a worse outcome in stroke patients treated with intravenous thrombolysis. Diabetes Care.

[16] Kim JT, Jahan R, Saver JL (2016). Impact of glucose on outcomes in patients treated with mechanical thrombectomy: a post hoc analysis of the solitaire flow restoration with the intention for thrombectomy study. Stroke.

[17] Paciaroni M, Agnelli G, Corea F, Ageno W, Alberti A, Lanari A (2008). Early hemorrhagic transformation of brain infarction: rate, predictive factors, and influence on clinical outcome: results of a prospective multicenter study. Stroke.

[18] Sacks DB (2013). Hemoglobin A1cin diabetes: panacea or pointless?. Diabetes.

[19] Kamouchi M, Matsuki T, Hata J, Kuwashiro T, Ago T, Sambongi Y (2011). Prestroke glycemic control is associated with the functional outcome in acute ischemic stroke: the Fukuoka stroke registry. Stroke.

[20] Murros K, Fogelholm R, Kettunen S, Vuorela AL, Valve J (1992). Blood glucose, glycosylated haemoglobin, and outcome of ischemic brain infarction. J Neurol Sci.

[21] Jing J, Pan Y, Zhao X, Zheng H, Jia Q, Li H (2016). Prognosis of ischemic stroke with newly diagnosed diabetes mellitus according to hemoglobin A1c criteria in Chinese population. Stroke.

[22] Robbins NM, Swanson RA (2014). Opposing effects of glucose on stroke and reperfusion injury: acidosis, oxidative stress, and energy metabolism. Stroke.

[23] van Seeters T, Biessels GJ, Kappelle LJ, van der Graaf Y, Velthuis BK (2016). Dutch acute stroke study (DUST) investigators. Determinants of leptomeningeal collateral flow in stroke patients with a middle cerebral artery occlusion. Neuroradiology.

[24] Reeson P, Jeffery A, Brown CE (2016). Illuminating the effects of stroke on the diabetic brain: insights from imaging neural and vascular networks in experimental animal models. Diabetes.

[25] Caplan LR (2015). Lacunar infarction and small vessel disease: pathology and pathophysiology. J Stroke.

[26] Lindsberg PJ, Roine RO (2004). Hyperglycemia in acute stroke. Stroke.

[27] Capes SE, Hunt D, Malmberg K, Pathak P, Gerstein HC (2001). Stress hyperglycemia and prognosis of stroke in nondiabetic and diabetic patients: a systematic overview. Stroke.

[28] Marik PE, Bellomo R (2013). Stress hyperglycemia: an essential survival response!. Crit Care.

[29] Marso SP, Daniels GH, Brown-Frandsen K, Kristensen P, Mann JF, Nauck MA (2016). Liraglutide and cardiovascular outcomes in type 2 diabetes. N Engl J Med.

[30] Marso SP, Bain SC, Consoli A, Eliaschewitz FG, Jódar E, Leiter LA (2016). Semaglutide and cardiovascular outcomes in patients with type 2 diabetes. N Engl J Med.

[31] Hong J, Zhang Y, Lai S, Lv A, Su Q, Dong Y (2012). Effects of metformin versus glipizide on cardiovascular outcomes in patients with type 2 diabetes and coronary artery disease. Diabetes Care.

[32] Park JM, Kang K, Cho YJ, Hong KS, Lee KB, Park TH (2016). Comparative effectiveness of Prestroke aspirin on stroke severity and outcome. Ann Neurol.

[33] Ishikawa H, Wakisaka Y, Matsuo R, Makihara N, Hata J, Kuroda J (2016). Influence of statin pretreatment on initial neurological severity and short-term functional outcome in acute ischemic stroke patients: the Fukuoka stroke registry. Cerebrovasc Dis.

