# Supplementary figures and images for: Interactive effect of acute and chronic glycemic indexes for severity in acute ischemic stroke patients
Source: BMC Neurol. 2018 Aug 3;18:105. doi: 10.1186/s12883-018-1109-1 (PMC6091005; doi:10.1186/s12883-018-1109-1)

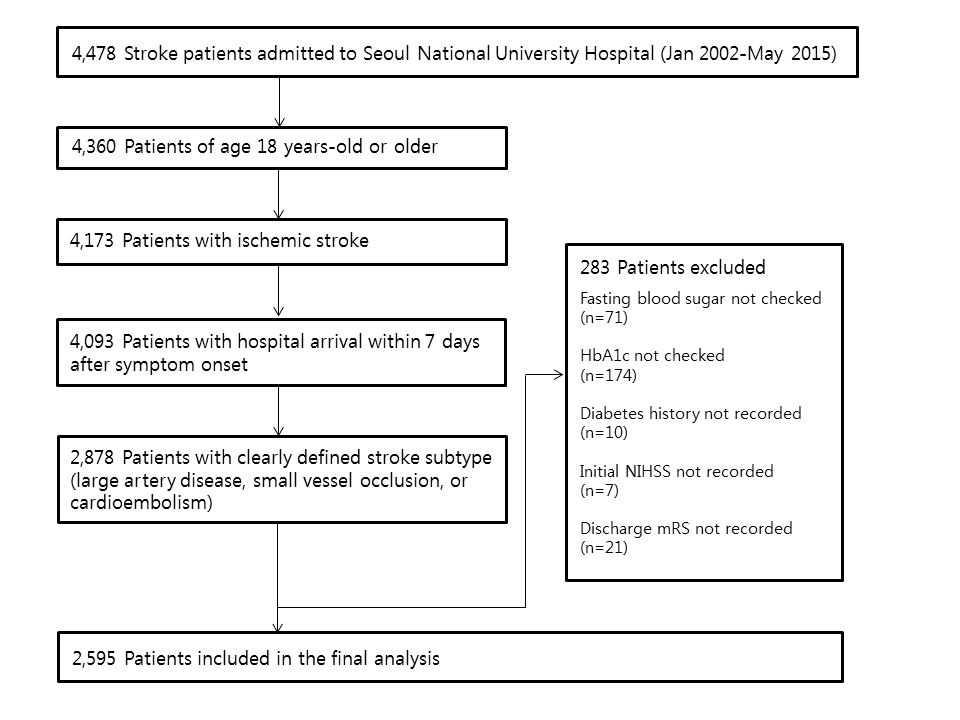

Supplement: Supplementary file 1 — Figure S1. Eligibility criteria of the study subjects. (TIF 86 kb) [file 12883_2018_1109_MOESM1_ESM.tif]

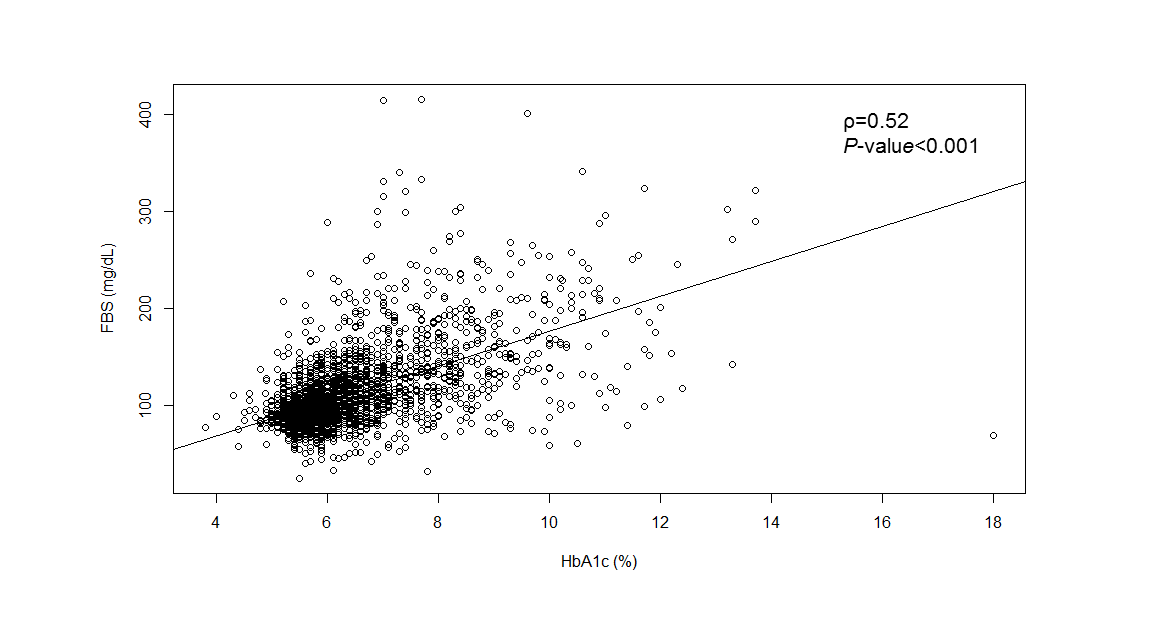

Supplement: Supplementary file 3 — Figure S2. Correlation plot between HbA1c and fasting blood sugar. (TIF 72 kb) [file 12883_2018_1109_MOESM3_ESM.tif]
