# Supplementary material for: Interactive effect of acute and chronic glycemic indexes for severity in acute ischemic stroke patients
Source: BMC Neurol. 2018 Aug 3;18:105. doi: 10.1186/s12883-018-1109-1 (PMC6091005; doi:10.1186/s12883-018-1109-1)
Supplement: Supplementary file 2 — Table S1. Interaction between glycemic parameters for functional outcome (modified Rankin’s scale) at discharge. Table S2. Interaction between glycemic parameters and stroke subtypes for initial stroke severity. (DOCX 25 kb) [file 12883_2018_1109_MOESM2_ESM.docx]

Table S1. Interaction between glycemic parameters for functional outcome (modified Rankin’s scale) at discharge

|  | Coefficient | Standard error (ε) | P-value |
| --- | --- | --- | --- |
| Model 1* |  |  |  |
| Initial NIHSS score | 0.32 | 0.01 | <0.001 |
| FBS (mg/dL) | 0.001 | 0.001 | 0.29 |
| HbA1c (%) | 0.02 | 0.05 | 0.63 |
| FBS * HbA1c | 0.0003 | 0.0005 | 0.51 |
| Model 2†* |  |  |  |
| FBS (mg/dL) | 0.007 | 0.001 | <0.001 |
| HbA1c (%) | 0.0002 | 0.04 | 0.996 |
| FBS * HbA1c | -0.001 | 0.0005 | 0.04 |
| Model 3** |  |  |  |
| Initial NIHSS score | -0.36 | 0.02 | <0.001 |
| FBS (mg/dL) | 0.0002 | 0.002 | 0.91 |
| HbA1c (%) | -0.03 | 0.06 | 0.63 |
| FBS * HbA1c | -0.001 | 0.0007 | 0.13 |
| Model 4†** |  |  |  |
| FBS (mg/dL) | -0.006 | 0.002 | <0.001 |
| HbA1c (%) | -0.001 | 0.05 | 0.98 |
| FBS * HbA1c | 0.0004 | 0.0006 | 0.49 |

NIHSS, National Institute of Health stroke scale; FBS, fasting blood sugar

* Shift analysis using a multivariable ordinal regression model

** Multivariable binary logistic regression model; the discharge modified Rankin’s scale score was dichotomized into good (0 to 1) and poor (2 to 6)

† Not including the initial NIHSS score in the model

All models were adjusted for age, sex, time to hospital arrival, body-mass index, stroke subtype, hypertension, hyperlipidemia, heart disease, previous stroke history, smoking, systolic and diastolic blood pressure, LDL cholesterol, HDL cholesterol, triglyceride level, acute antithrombotics use (antiplatelet and anticoagulants), and hyperacute reperfusion therapy

Table S2. Interaction between glycemic parameters and stroke subtypes for initial stroke severity

|  | Coefficient (B) | Standard error (ε) | t-value | P-value |
| --- | --- | --- | --- | --- |
| FBS (mg/dL) | 0.04 | 0.005 | 7.57 | <0.001 |
| HbA1c (%) | -0.31 | 0.17 | -1.84 | 0.07 |
| TOAST classification |  |  |  |  |
| LAD (reference) | - | - | - | - |
| SVO. | -1.92 | 0.25 | -7.57 | <0.001 |
| CE | 2.01 | 0.35 | 5.77 | <0.001 |
| FBS * HbA1c | -0.008 | 0.002 | -3.63 | <0.001 |
| FBS * SVO | -0.04 | 0.01 | -4.96 | <0.001 |
| FBS * CE | 0.02 | 0.01 | 2.28 | 0.02 |
| HbA1c * SVO | 0.54 | 0.23 | 2.38 | 0.02 |
| HbA1c * CE | -0.41 | 0.25 | -1.62 | 0.10 |
| FBS * HbA1c * SVO | 0.008 | 0.003 | 2.38 | 0.01 |
| FBS * HbA1c * CE | -0.01 | 0.004 | -2.68 | 0.007 |

FBS, fasting blood sugar; LAD, large artery disease; SVO, small vessel occlusion; CE, cardioembolism

Adjusted for age, sex, time to hospital arrival, body-mass index, hypertension, hyperlipidemia, heart disease, previous

stroke history, smoking, systolic and diastolic blood pressure, LDL cholesterol, HDL cholesterol, and triglyceride level
